# Semi-Targeted Profiling of Bile Acids by High-Resolution Mass Spectrometry in a Rat Model of Drug-Induced Liver Injury

**DOI:** 10.3390/ijms24032489

**Published:** 2023-01-27

**Authors:** Myriam Mireault, Vivaldy Prinville, Leanne Ohlund, Lekha Sleno

**Affiliations:** Department of Chemistry, Université du Québec à Montréal (UQAM), P.O. Box 8888, Downtown Station, Montreal, QC H3P 3C8, Canada

**Keywords:** acetaminophen, bile acids, hepatotoxicity, liquid chromatography–high resolution mass spectrometry, metabolomics, rat plasma, drug-induced liver injury

## Abstract

Using a semi-targeted approach, we have investigated the effect of acetaminophen on circulating bile acid profiles in rats, including many known bile acids and potential isomeric structures, as well as glucuronide and sulfate conjugates. The chromatographic separation was based on an optimized reverse-phase method exhibiting excellent resolution for a complex mix of bile acids using a solid-core C18 column, coupled to a high-resolution quadrupole time-of-flight system. The semi-targeted workflow consisted of first assigning all peaks detectable in samples from 46 known bile acids contained in a standard mix, as well as additional peaks for other bile acid isomers. The presence of glucuronide and sulfate conjugates was also examined based on their elemental formulae and detectable peaks with matching exact masses were added to the list of features for statistical analysis. In this study, rats were administered acetaminophen at four different doses, from 75 to 600 mg/kg, with the highest dose being a good model of drug-induced liver injury. Statistically significant changes were found by comparing bile acid profiles between dosing levels. Some tentatively assigned conjugates were further elucidated using in vitro metabolism incubations with rat liver fractions and standard bile acids. Overall, 13 identified bile acids, 23 tentatively assigned bile acid isomers, and 9 sulfate conjugates were found to increase significantly at the highest acetaminophen dose, and thus could be linked to drug-induced liver injury.

## 1. Introduction

Bile acids (BA) are synthesized from cholesterol in the liver through a series of enzymatic reactions and then modified by gut bacteria [1]. All BA structures are based on a lipophilic steroidal core with at least one hydroxyl group and a carboxylate side chain which can be conjugated to glycine or taurine. Bile acids are a very diverse class of metabolites, with a wide range of polarities. This is a determining factor for the function and toxicity of different BAs in the body [1,2].

Bile acids have several physiological functions. They serve as detergents for the absorption and digestion of lipids, cholesterol, and fat-soluble vitamins, by forming micelles in the intestinal lumen [1,2]. They are also signaling molecules in the immune response, as well as in the maintenance of glucose and lipid homeostasis [1,3,4]. Bile acids have exhibited a protective role in neurodegenerative diseases, such as Alzheimer’s, Parkinson’s, and Huntington’s diseases, as well as amyotrophic lateral sclerosis [3]. However, high BA concentrations can be toxic and cause liver damage. Depending on their physicochemical properties, some BAs, generally those with higher hydrophobicity, can induce oxidative stress, apoptosis, necrosis, and a pro-inflammatory response [2,5,6]. Certain biotransformation reactions, such as sulfation and glucuronidation which increase their polarities, can reduce the toxicity of BAs. These phase II conjugation reactions transfer a sulfate or glucuronide group to the BA which increases their elimination from the body [7,8].

The circulation of BAs depends on the enterohepatic flow. Primary bile acids are synthesized in hepatocytes and include cholic acid (CA) and chenodeoxycholic acid (CDCA) in humans, whereas in rats, CDCA is converted to *α*-muricholic acid (*α*-MCA) and *β*-muricholic acid (*β*-MCA). They are usually conjugated to glycine or taurine prior to being secreted into the bile. Then, they are secreted into the intestine where they are deconjugated and transformed into secondary BAs. Approximately 95% of BAs are reabsorbed in the liver and excreted in the bile to complete the enterohepatic circulation [2,3]. A hepatic lesion, even minor, can disrupt this flow and lead to an increase in BAs in the blood [9]. Thus, these molecules have the potential to be sensitive biomarkers to detect liver damage [9,10,11,12]. However, liver damage can be caused by a variety of factors, including drug-induced liver injury (DILI) [4,9]. Thus, it is important to understand the effect of DILI on circulating bile acids.

Acetaminophen (APAP) is an analgesic commonly used worldwide to relieve pain and reduce fever [13]. Unfortunately, when taken in excess or under oxidative stress, this drug can cause acute liver failure through the formation of its reactive metabolite, N-acetyl p-benzoquinone imine (NAPQI) [13,14]. At therapeutic doses, approximately 85% of APAP is metabolized into phase II glucuronide or sulfate conjugates [13,14], and 5 to 10% undergoes oxidation mainly by CYP2E1 to form NAPQI [13,14,15]. NAPQI is often conjugated with glutathione (GSH) which traps its reactive group and eventually gets further metabolized into mercapturic acid conjugate for elimination in urine [13,16,17]. Consumption above the maximum recommended dose (3–4 g/day) [18] results in saturation of sulfation and glucuronidation pathways, and increases NAPQI formation [13], in turn depleting GSH reserves and promoting covalent modification of liver proteins [14,17].

Acetaminophen is the main cause of acute liver failure (ALF) in the Western world [19,20]. To treat ALF, it is necessary to act quickly before hepatotoxicity has progressed too far. To date, the only treatment is N-acetylcysteine (NAC) [21], to trap excess NAPQI and help restore glutathione levels [13,21]. Administration of NAC is usually necessary within 8 h of APAP overdose to prevent irreversible liver damage [21,22]. Indeed, studies have shown that high doses of APAP influence the levels of some bile acids [9,10,23]. A better understanding of this effect is warranted, and specific BAs could be useful as biomarkers of the severity of liver damage.

Several analytical methods have been reported for the detection and quantitation of bile acids, including liquid chromatography–mass spectrometry (LC-MS/MS) which offers high sensitivity and potential for discriminating between very similar structures in complex biological samples [24]. Many studies have used a targeted approach based on multiple reaction monitoring (MRM) as a selective method for accurate quantification [23,24,25,26]. However, this type of analysis is usually limited to available bile acid standards. 

In this study, a semi-targeted analysis using high-resolution tandem mass spectrometry was used to expand on the number of BAs found to have significant changes, and thus better understand the effect of APAP on BA profiles in rats. This method allows not only the investigation of changes in known structures, using a standard mix of 46 BAs, but several others, including isomeric species, as well as glucuronides and sulfate conjugates formed via further metabolism of bile acids. The present study gives a more comprehensive picture since it adds many isomeric bile acids as well as sulfate and glucuronide conjugates to the list of features studied in the context of differential changes with increasing APAP dose. First, we verified that our semi-targeted approach detected the same BAs from the standard mix in the rat samples as the previous study and that the changes induced by APAP were the same. Then, we studied the potential isomers of these BAs as well as their conjugated forms to sulfate or a glucuronic acid to better understand the impact of APAP on the metabolism of BAs. For specific isomeric and sulfate conjugates found to increase significantly between low and high APAP doses, in vitro incubations were used to better assign their structures.

## 2. Results

### 2.1. Assigned Bile Acids

A semi-targeted analysis was used to investigate bile acid profiles in rat plasma following the administration of increasing APAP doses. The chromatographic separation used was based on a method previously developed in our laboratory for the targeted LC-MRM analysis of forty-six BAs [23]. In the present study, mass spectrometry data acquisition was performed using a high-resolution quadrupole time-of-flight (Qq-TOF) system to enable a more complete picture of bile acid profiles and the effect of four different doses of APAP.

**Figure 1:**
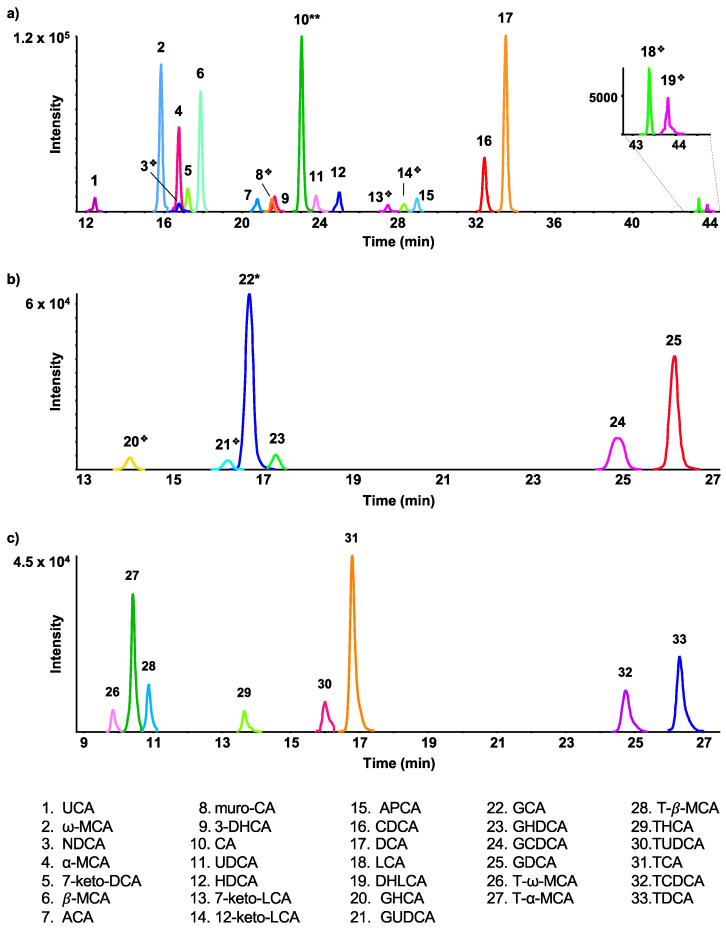
Extracted ion LC-MS chromatograms, in negative ion mode, of 33 known bile acids well detected in rat plasma 24 h following 600 mg/kg APAP dose, including unconjugated bile acids (**a**), glycine conjugated bile acids (**b**), and taurine-conjugated bile acids (**c**). The peaks were scaled and annotated as follows: * decreased 2×, ** decreased 6×, and ❖ increased 5×, for clarity.

Detectable peaks in plasma samples were first assigned based on an exact mass and retention time comparison with 46 bile acid standards. At the highest dose of 600 mg/kg APAP, thirty-three plasma BAs (Figure 1) could be positively identified, compared to 39 BAs having been previously assigned by our targeted LC-MRM method [23]. Of the six BAs not detected here, five peaks (NUDCA, GLCA, di-oxo-LCA, 6,7-diketo-LCA, and NCA) showed low signal by LC-MRM with no significant changes between different APAP doses [23]. The advantage of LC-MRM analysis is the ability to optimize the detection sensitivity of each targeted analyte, with separate optimal collision energies used for each transition. However, in the case of unconjugated bile acids, due to their very limited fragmentation, MRM transitions used for these species are often precursor to precursor ions, which, at low mass resolution, have the potential to exhibit interferences in complex biological samples. Iso bile acids, such as IDCA, AILCA, and ILCA, were not detected in the plasma samples, as they are mainly found in feces [27]. DHCA is predominantly converted to 3-*α*-hydroxylated-oxo bile acids to regulate BA flow. Only a small portion of DHCA is secreted in its unchanged form [28], thus preventing its detection and that of its conjugates (GDHCA and TDHCA) (Figure 1).

**Figure 2:**
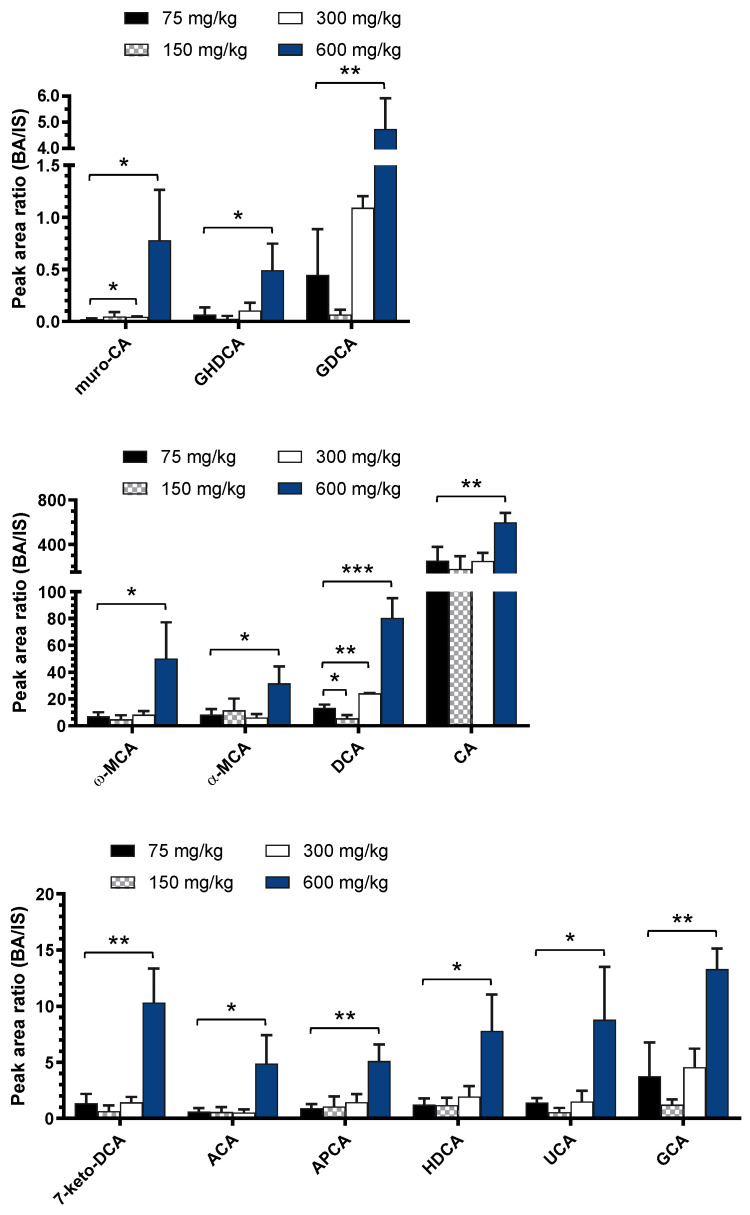
Peak area ratios of assigned standard bile acids having significant changes between 75 mg/kg and 600 mg/kg APAP dosing. Statistical significance is shown with *p*-values <0.05 (*), <0.01 (**), or <0.001 (***).

By comparing low- and high-dose groups, statistically significant changes were found with area ratios normalized using deuterated internal standards (Table S1) for 13 BAs from the standard mix (Figure 2, Table S2). Three glycine-conjugated BAs (GCA, GDCA, and GHDCA) increased significantly, whereas no taurine-conjugated BAs were shown to vary significantly. In general, taurine-conjugated bile acids predominate over glycine-conjugated ones in rats [29]. However, when APAP is taken in excess, GSH reserves are depleted [14], and since glutathione and taurine are biosynthesized using the same precursor, cysteine, this could hinder the synthesis of taurine and thus reduce its levels in the body [9]. Our results, consistent with those of Yamazaki et al. [9], show that APAP can interfere with conjugation pathways that reduce BA toxicity and promote the conjugation of BAs with glycine.

Deoxycholic acid (DCA) shows a highly significant increase between the two doses with a *p*-value < 0.001 with a fold change of 6.0 (Figure 2, Table S2). It is a highly hydrophobic BA that can inhibit cell proliferation by stimulating the farnesoid X receptor (FXR) [30] and induce cell apoptosis [31]. Hyodeoxycholic acid (HDCA), which is also significantly increased (*p*-value 0.019 and fold change of 6.4), plays a role in inhibiting cell proliferation as well. It acts not only through the FXR pathway, but also reduces levels of BAs involved in cell proliferation [32]. Cholic acid (CA) is very abundant in rat plasma [29], unlike ACA, a fetal BA that is not usually present in adult rats [33]. Nevertheless, our results show a significant increase in these two molecules. Interestingly, studies have shown that the increase in CA and ACA in bile correlate with liver regeneration, thus suggesting their involvement in this process [33,34]. Indeed, Bhushan et al. showed that CA administration plays an important role in liver regeneration [35]. Interestingly, some BAs decrease between doses of 75 and 150 mg/kg APAP, followed by an increase at 300 mg/kg APAP (Figure 2). However, this decrease is not always significant.

### 2.2. Putatively Identified Bile Acid Isomers

**Figure 3:**
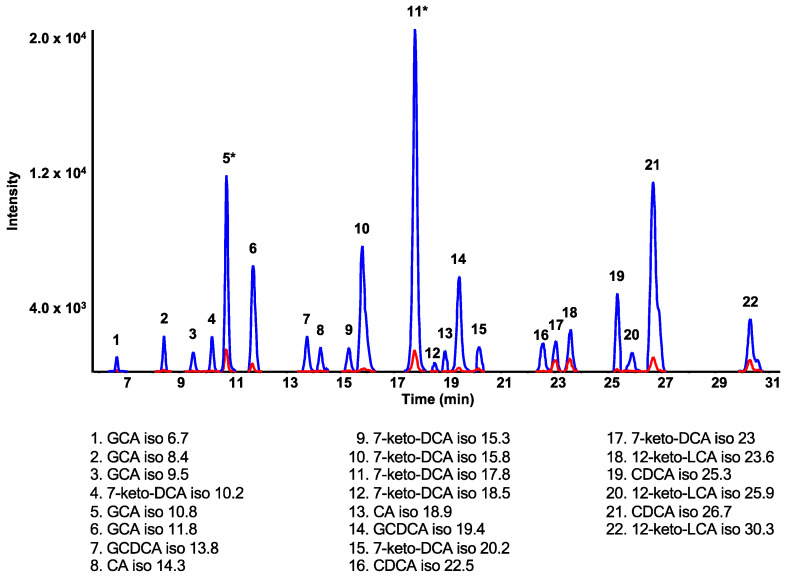
Extracted ion LC-MS chromatograms, in negative ion mode, of tentatively assigned bile acid isomers having significant changes between low and high APAP dosing. Peaks from a rat treated with 75 and 600 mg/kg APAP are shown in red or blue, respectively. Peaks annotated with * were decreased by 4×.

The enterohepatic cycle allows the synthesis and recycling of bile acids. It can occur 10 times a day forming a plethora of BA structures, including several isomeric species [2,36]. For example, CDCA and UDCA have the same structure, except for the orientation of their C7 hydroxyl group (CDCA is *α*-oriented, and UDCA is *β*-oriented), imparting different biological roles [2]. Taking advantage of the high-resolution data acquired in an untargeted manner, a larger number of BAs were accessible for this analysis. Therefore, potential isomers of BAs not contained in the standard mixture could also be investigated for their changing levels with increasing APAP dose. Twenty-two tentatively identified isomers were shown to increase significantly between the lowest and highest doses of APAP administered (Figure 3, Table 1), with five of them having *p*-values less than 0.01. A keto-DCA isomer (elemental formula corresponding to C_24_H_38_O_5_) eluting at 15.8 min corresponds to the isomer with the lowest *p*-value (0.0018) followed by the GCDCA isomer (C_26_H_43_NO_5_) at 19.4 min with a *p*-value of 0.0026, both of which had large changes in peak area ratios of 12- and 8-fold, respectively (Figure 3, Table 1).

### 2.3. Glucuronide- and Sulfate-Conjugated Bile Acids

Sulfate and glucuronide conjugates of bile acids were also investigated to better understand the potential perturbations in bile acid metabolism involved in drug-induced liver injury. These are two phase II conjugation reactions that increase the solubility of bile acids, promoting their elimination in urine or bile [7,8]. Putative conjugates of the bile acids were assessed by accurate mass filtering. Their structures, however, were not confirmed, and the names assigned here correspond to a standard BA identified within the standard mix, however it can be any isomer of that formula.

**Figure 4:**
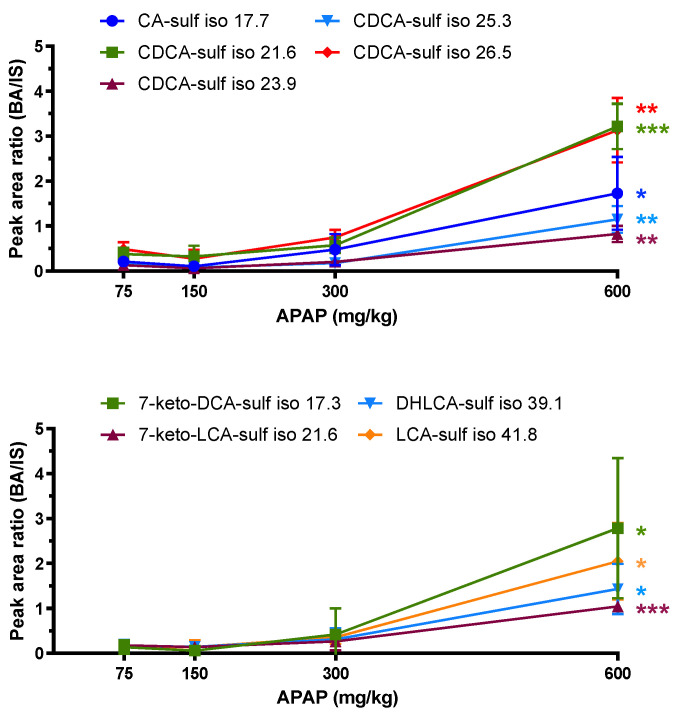
Peak area ratios of tentatively assigned sulfate conjugates having significant changes between 75 and 600 mg/kg APAP dose. Statistical significance is shown with *p*-value <0.05 (*), <0.01 (**), or <0.001 (***).

Six putative glucuronide and thirteen sulfate conjugates were detected in plasma from rats receiving the highest APAP dose. Of these, nine sulfate-conjugated species increased significantly between the lowest and highest doses (Figure 4, Table 2). Four CDCA-sulfate isomers eluting at 21.6, 23.9, 25.3, and 26.5 min showed significant changes, with the earliest eluting peak having a *p*-value less than 0.001, as did a keto-LCA-sulfate isomer (Figure 4, Table 2). Keto-LCA and CDCA are mono-OH and di-OH bile acids, respectively, making them more hydrophobic and potentially more toxic than tri-OH bile acids, such as CA [2]. They are therefore more susceptible to sulfation than those with multiple hydroxyl groups [29].

No glucuronide conjugates were found to vary significantly with increased APAP dose. In rats, glucuronidation and sulfation of BAs are usually minor pathways compared to glycine or taurine conjugates [29]. Nevertheless, our results show that when exposed to high doses of APAP, the sulfation pathway could help compensate for the accumulation of more hydrophobic BAs.

### 2.4. In Vitro Metabolism Incubations

A large variety of bile acids are present in biological samples, including many isomeric species. Therefore, obtaining all possible bile acid standards is not very feasible. To investigate further some of the putatively identified bile acid isomers and conjugates of specific interest, in vitro incubations were performed with rat liver microsomes and metabolism cofactors. The metabolites of nine bile acids (CA, *α*-MCA, *β*-MCA, CDCA, DCA, UDCA, LCA, GCA, and GCDCA) were studied under oxidative and sulfation conditions (Figure S1). CA, *α*-MCA, and *β*-MCA bile acids are three isomers with the formula C_24_H_40_O_5_. CA incubations formed three oxidized metabolites, including two CA+O (hydroxylated CA) and one CA+O-2H (keto-CA). *α*-MCA formed four hydroxylated metabolites and one keto-MCA (*α*-MCA+O-2H), while *β*-MCA formed two hydroxylated and one keto metabolite (Figure S1a–c). CDCA, DCA, and UDCA are all di-OH Bas with a formula of C_24_H_40_O_4_. CDCA formed a hydroxylated metabolite (CDCA+O) as well as a sulfated metabolite (CDCA+SO_3_), while DCA formed seven hydroxylated species, one dehydroxylated metabolite, one keto-DCA, and one sulfate conjugate. UDCA formed five hydroxylated species and one sulfated metabolite (Figure S1d–f). LCA, a mono-hydroxylated BA, formed the most metabolites, including six dihydroxylated and four mono-hydroxylated metabolites, one keto form, and one sulfate conjugate (Figure S1i). The two tested glycine-conjugated acids also formed metabolites following in vitro incubations (Figure S1g,h). For GCA (C_26_H_43_NO_6_), one keto metabolite (GCA+O-2H) was detected, while GCDCA (C_26_H_43_NO_5_) formed twelve oxidized metabolites (seven GCDCA+O, four GCDCA+O-2H, and one GCDCA+2O) (Figure S1g,h).

**Figure 5:**
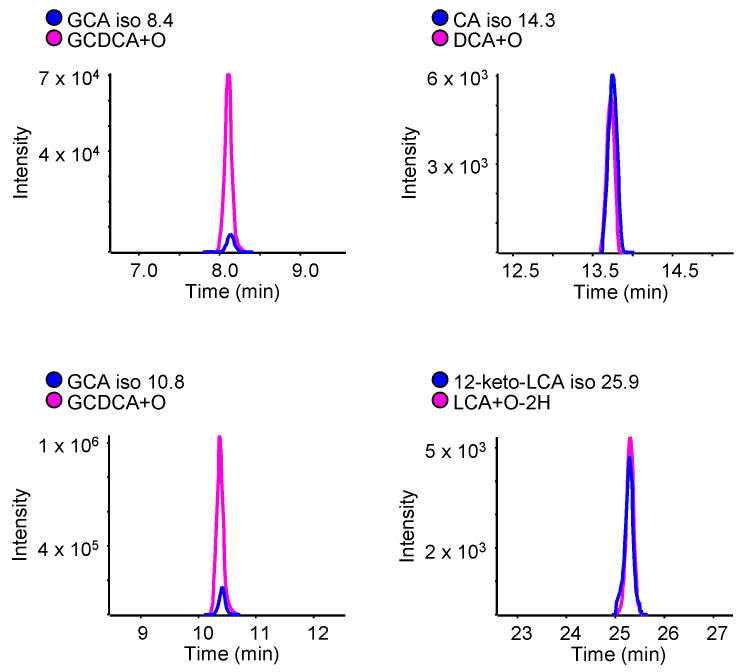
High-resolution extracted ion LC-MS chromatograms of isomers overlaid from rat plasma samples (in blue) and in vitro incubation samples with standard bile acids (in pink).

Peaks from metabolites detected in these incubations were compared with putatively assigned isomers and their conjugates shown to be significantly increased in rat samples from the 600 mg/kg APAP group. Unfortunately, none of the peaks corresponding to sulfated BAs of interest corresponded to the same retention times as those found in in vitro incubations, but four putatively assigned BA isomers were identified, based on retention time matching with metabolites formed under oxidative conditions (Figure 5). Even though exact structures cannot be confirmed from this data, this brings us closer to assigning certain tentative BA structures perturbed in the context of drug-induced liver injury.

## 3. Discussion

Bile acids are a diverse class of molecules, and disruption of the enterohepatic flow has been shown to correlate with DILI [1,9]. However, previous studies have focused on specific known BAs to study the impact of drugs [9,10,11,12,37]. In this study, a semi-targeted approach was taken to investigate the effect of increasing APAP on rat bile acid profiles in a more comprehensive manner. A treatment of 600 mg/kg was used as the highest dose administered, because it starts showing effects of hepatoxicity in rats and was previously shown to drastically increase NAPQI-adducted protein in rat plasma, in contrast to the 75 mg/kg treatment [38]. The purpose of this study was to compare non-toxic and toxic APAP consumption. High doses of APAP increased not only the levels of BAs associated with hepatotoxicity, but also those with a protective role. CA and ACA are two tri-OH BAs involved in liver regeneration [33,34], while DCA and HDCA (di-OH) have been shown to inhibit cell proliferation [31,32]. Moreover, DCA has a role in apoptosis [31]. The toxicity induced by BAs depends on their hydrophobicity, where a mono-OH exhibits greater toxicity than a di-OH [2]. They can be conjugated (through amidation with glycine or taurine, or via sulfation or glucuronidation) to increase their polarity and promote their elimination [7,8,39,40]. In rats, amidation is the major pathway, with taurine conjugation usually predominating over glycine conjugation [29]. However, excessive APAP consumption can interfere with taurine synthesis and instead promote glycine conjugation [9]. Sulfation is generally a minor pathway of elimination for increasing the polarity of hydrophobic BAs. Thus, mono-hydroxylated BAs are more conjugated to sulfate than di- and tri-hydroxylated ones [29]. Our results show that the APAP-induced increase in the levels of CA, DCA, HDCA, as well as CA isomers also leads to an increase in the corresponding glycine conjugates (Figure 2 and Figure 3, Table 1 and Table S2), while many CDCA isomers have increasing sulfation pathways (Figure 3 and Figure 4, Table 1 and Table 2).

Hepatic fractions are commonly used to study phase I and II biotransformations to better understand compound metabolism and toxicity [41]. For example, Thakare et al. [42] used hepatic S9 fractions to characterize the metabolism of four BAs (LCA, UDCA, CDCA, and CA) and compare their metabolic pathways between different species. In the current study, in vitro incubations were used to help identify BAs with that changed significantly between low and high APAP doses. Bile acid metabolites are not all commercially available, therefore in vitro incubations can serve as an alternative allowing for a better assignment of these metabolites. We incubated nine BA standards (CA, *α*-MCA, *β*-MCA, CDCA, DCA, UDCA, LCA, GCA, and GCDCA), provided a better assignment of four BA isomers of specific interest in the context of this study, in addition to characterizing many different metabolites from these bile acids that will be useful for future studies (Figure 5 and Figure S1).

A significant challenge is the structural elucidation of unknown BAs, due to limited structural information from MS/MS fragmentation. Fortunately, the glycine, taurine, and sulfate conjugates can be easily distinguished due to their specific fragments (Tables S4 and S5). Glycine conjugates have a specific fragment at *m/z* 74.02, with good intensity in their MS/MS spectra, corresponding to deprotonated glycine, while taurine conjugates have characteristic fragments at *m/z* 124.01 (taurine), 106.98 (C_2_H_3_SO_3_^−^), 80.96 (HSO_3_^−^), and 79.96 (SO_3_^−^ radical). Sulfate conjugates also showed a characteristic product ion at *m/z* 96.96 corresponding to the HSO_4_^−^ ion. These characteristic fragments have been verified to help confirm tentatively assigned structures. In one instance, for example, a bile acid initially assigned by exact mass as a sulfate conjugate of a GLCA isomer (at a retention time of 10.9 min), was rather a taurine conjugate based on its MS/MS spectrum with the characteristic fragment ion at *m/z* 124.01. A possibility with the same elemental formula would be a taurine conjugate of a keto DCA isomer. Unconjugated bile acids do not fragment well (Tables S3 and S5), with most fragments having very low intensities (<1%), common to different bile acids due to the structural similarity of these metabolites and their steroidal nuclei. In addition, in the case of peaks with low intensity, there is a large chance of product ions coming from background signals, reducing the ability to identify the unconjugated BAs of interest (Figure 5).

## 4. Materials and Methods

### 4.1. Chemicals and Reagents

4-Acetamidophenol (acetaminophen, APAP, 98%), cholic acid (CA), chenodeoxycholic acid (CDCA), glycocholic acid (GCA), glycochenodeoxycholic acid (GCDCA), deoxycholic acid (DCA), lithocholic acid (LCA), taurocholic acid (TCA), taurodeoxycholic acid (TDCA), taurolithocholic acid (TLCA), ursodeoxycholic acid (UDCA), uridine-5′-diphosphoglucuronic acid (UDPGA), 3′-phosphoadenosine-5′-phosphosulfate (PAPS), nicotinamide adenine dinucleotide phosphate (NADP^+^), glucose-6-phosphate, MgCl_2_, glucose-6-phosphate dehydrogenase, acetonitrile (ACN), and methanol (MeOH) (both HPLC grade) as well as formic acid (LC-MS grade) were all purchased from Sigma-Aldrich (Oakville, ON, Canada). Rat (Sprague−Dawley) liver microsomes (RLM, part #452501) and S9 hepatic fractions (RS9, part #452591) were purchased from Corning (Corning, NY, USA). Ultrapure water was from a Millipore Synergy UV system (Billerica, MA, USA). MetaboloMetrics^TM^ bile acid analysis kits contained a standard mix of 46 bile acids and 14 deuterated isotope-labeled internal standards and were obtained from MRM Proteomics Inc. (Montreal, QC, Canada). Sprague Dawley rats were treated by intraperitoneal injection with 75, 150, 300, and 600 mg/kg APAP in triplicate. Rat plasma was collected after 24 h at the INRS Centre de Biologie Expérimentale (Laval, QC, Canada), within standard ethical practices of the Canadian Council on Animal Care (project UQLK.14.02). These samples were collected in February 2014 and stored at −80 °C until proceeding with sample preparation.

A standard mix of 46 bile acids was provided as a dried sample (tube A). Bile acids were present at a concentration of 2.5 nmol, except for deoxycholic acid (DCA) at 5 nmol and taurohyocholic acid (THCA) at 6.5 nmol. The bile acids in the standard mix were as follows: 12-ketodeoxycholic acid (12-keto-DCA), 12-ketolithocholic acid (12-keto-LCA), 3-dehydrocholic acid (3-DHCA), 7-ketodeoxycholic acid (7-keto-DCA), 7-ketolithocholic acid (7-keto-LCA), allocholic acid (ACA), alloisolithocholic acid (AILCA), apocholic acid (APCA), chenodeoxycholic acid (CDCA), cholic acid (CA), dehydrocholic acid (DHCA), deoxycholic acid (DCA), dioxolithocholic acid (di-oxo-LCA), glycochenodeoxycholic acid (GCDCA), glycocholic acid (GCA), glycodeoxycholic acid (GDCA), glycohyocholic acid (GHCA), glycohyodeoxycholic acid (GHDCA), glycolithocholic acid (GLCA), glycoursodeoxycholic acid (GUDCA), hyodeoxycholic acid (HDCA), isodeoxycholic acid (IDCA), isolithocholic acid (ILCA), lithocholic acid (LCA), murocholic acid (muro-CA), norcholic acid (NCA), nordeoxycholic acid (NDCA), norursodeoxycholic acid (NUDCA), tauro-*α*-muricholic acid (*α*-TMCA), tauro-*β*-muricholic acid (*β*-TMCA), tauro-ω-muricholic acid (ω-TMCA), taurochenodeoxycholic acid (TCDCA), taurocholic acid (TCA), taurodehydrocholic acid (TDHCA), taurodeoxycholic acid (TDCA), taurohyocholic acid (THCA), taurolithocholic acid (TLCA), tauroursodeoxycholic acid (TUDCA), ursocholic acid (UCA), ursodeoxycholic acid (UDCA), *α*-muricholic acid (*α*-MCA), *β*-muricholic acid (*β*-MCA), ω-muricholic acid (ω-MCA), 6,7-diketolithocholic acid (6,7-diketo-LCA), dehydrolithocholic acid (DHLCA), and glycodehydrocholic acid (GDHCA).

A mix of isotopically labeled bile acids (0.1–0.75 nmol) was provided as a dried sample (tube B) and was used as internal standard for data normalization. The labeled bile acids in the internal standard mix were as follows: glycoursodeoxycholic acid-d_4_ (d_4_-GUDCA), glycocholic acid-d_4_ (d_4_-GCA), tauroursodeoxycholic acid-d_4_ (d_4_-TUDCA), taurocholic acid-d_4_ (d_4_-TCA), cholic acid-d_4_ (d_4_-CA), ursodeoxycholic acid-d_4_ (d_4_-UDCA), glycochenodeoxycholic acid-d_4_ (d_4_-GCDCA), glycodeoxycholic acid-d_4_ (d_4_-GDCA), taurochenodeoxycholic acid-d_4_ (d_4_-TCDCA), taurodeoxycholic acid-d_6_ (d_6_-TDCA), chenodeoxycholic acid-d_4_ (d_4_-CDCA), deoxycholic acid-d_4_ (d_4_-DCA), glycolithocholic acid-d_4_ (d_4_-GLCA), and lithocholic acid-d_4_ (d_4_-LCA).

### 4.2. Sample Preparation

#### 4.2.1. Standard and Internal Standard Mix

For the standard MetaboloMetrics mix of 46 bile acids, 250 µL ACN was added to the lyophilized sample (tube A) and the solution was diluted 10-fold with 40% ACN, 60% water. For the internal standard mix, 7.5 mL of 40% ACN was added to tube B, and then vortexed, sonicated 15 min, and diluted 1:3 with ACN 40%.

#### 4.2.2. Extraction of Plasma Samples

Rat plasma (50 µL) samples were mixed with the internal standard solution (50 µL), followed by the addition of 300 µL MeOH to precipitate proteins. Samples were vortexed, sonicated for 15 min, and centrifuged at 14,000 rpm for 8 min at 4 °C. Supernatants (300 µL) were dried and reconstituted in 150 μL of 50% MeOH prior to LC-MS/MS analysis.

### 4.3. In Vitro Incubations

CA, *α*-MCA, *β*-MCA, CDCA, DCA, UDCA, LCA, GCA, or GCDCA (10 µM) were pre-incubated with rat S9 fractions (at 2 mg/mL protein) at 37 °C for 3 min in 100 mM phosphate buffer, pH 7.4. Then, 5 mM MgCl_2_, 10 mM glucose-6-phosphate, 0.5 mM NADP^+^, 1 mM PAPS, and 2 units/mL glucose-6-phosphate dehydrogenase were added. Samples (100 µL) were incubated at 37 °C for 1 h, and incubations without either NADP^+^ or PAPS added were used as controls to verify which peaks were related to metabolites from each cofactor. The reactions were quenched with cold ACN and centrifuged at 14,000 rpm for 8 min at 4 °C. Supernatants were dried and reconstituted in 100 µL 10% ACN for LC-MS/MS analysis.

### 4.4. LC-MS/MS Analysis

Liquid chromatography–high resolution tandem mass spectrometry analyses were performed on Shimadzu Nexera UHPLC coupled to a quadrupole time-of-flight system (TripleTOF^®^ 5600^+^, Sciex) (Concord, ON, Canada), equipped with a Duospray ion source operated in negative electrospray mode. Data were calibrated in TOF-MS and MS/MS mode automatically every four injections using a calibrant delivery system with a set of in-house standard compounds ranging from *m/z* 119–966. Bile acids were separated on an Aeris^TM^ Peptide XB-C18 column (100 × 2.1, 1.7 um) (Phenomenex, Torrance, CA) with gradient elution using water and ACN, both containing 0.1% formic acid as mobile phases A and B, respectively, at 0.25 mL/min with a column temperature of 40 °C. The gradient started at 10% B (held for 1.5 min), then increased linearly to 25% for 1.5 min, to 35% over 17 min, to 50% over 20 min, to 60% over 2 min, and 90% over 0.5 min, and held for 1 min, with a total run time of 55 min including column re-equilibration. The acquisition of the TOF-MS and MS/MS spectra was carried out with the following source parameters: ionization voltage at −4500 V, source temperature of 450 °C, curtain gas of 30 psi, drying and nebulizer gases at 50 psi, and declustering potential of 80 V. TOF-MS was collected from *m/z* 115–980 with an accumulation time of 300 ms, followed by MS/MS from *m/z* 50–900 on the top 5 ions using information-dependent acquisition (IDA) mode, with dynamic background subtraction. Each MS/MS acquisition had an accumulation time of 150 ms and a collision offset voltage of 30 ± 10 V.

### 4.5. Data Analysis

To normalize data, isotopically labeled bile acids were added to rat samples prior metabolite extraction. Internal standards for each bile acid peak were selected according to their structural similarity, their signal in the sample, and their peak shape, as well as retention time. The assignment of IS for each bile acid is summarized in Table S1. The standard mix of 46 bile acids was used to identify known compounds present in extracted plasma samples, based on accurate mass and retention time. Isomeric species were tentatively assigned based on accurate mass measurements (within 10 ppm) corresponding to identical elemental formulae as the standard bile acids but with different retention times. Then, the detection of glucuronide and sulfate conjugates of these same bile acids was carried out by accurate mass filtering (adding exact mass of glucuronide or sulfate conjugation). For in vitro incubations, metabolism samples were compared to controls to detect oxidative metabolites and sulfate conjugates. These metabolites were then compared with those present in plasma samples, based on accurate mass measurements (within 10 ppm) and retention time matching with metabolites formed in vitro. Data visualization and MS/MS spectra comparison (Tables S3–S5) were performed using Peak View^®^ 2.2 with MasterView^TM^ 1.1, and peak integration used MultiQuant^TM^ 2.1 (Sciex, Concord, ON, Canada). To identify changing features with APAP dose in rats, integration data was imported from MultiQuant^TM^ into MarkerView^TM^ 1.2.1 (Sciex) for statistical analyses. Area ratios (analyte/IS) for all compounds associated with standard bile acids, tentatively assigned isomers, and sulfate conjugates were compared using *t*-test analysis within MarkerView^TM^, and a threshold of *p*-value < 0.05 and a fold change over 2 was set as statistically significant.

## 5. Conclusions

The effect of increased APAP dose on bile acid profiles was investigated using high-resolution mass spectrometry, allowing the confirmation of 33 bile acids present in rat plasma, as well as 50 putatively assigned isomers and 19 phase II conjugates. From this complex data set, certain bile acids and isomers exhibited elevated levels as APAP dose increased. Of those having significant changes at the highest dose administered, 13 BAs had confirmed structures, with an additional 23 isomers and 9 sulfate conjugates being tentatively assigned. High doses of APAP were shown to favor conjugation with glycine over taurine, whereas increased sulfation could result as a detoxification mechanism for accumulated BAs. The eventual use of bile acids as biomarkers in humans to classify the severity of APAP-associated liver toxicity requires further investigation [25,29]. This study sheds light on the changes occurring in the complex metabolism of BAs in rats with four separate dosing levels in a more controlled study than what is possible in humans. BA profiles were most affected by the highest APAP dose administered, where hepatotoxicity becomes evident in rats. 

## Figures and Tables

**Table 1:** Tentatively assigned bile acid isomers with significant changes between low and high APAP doses.

Bile Acid	Formula	RT (min)	*p*-Value	Fold Change * (600/75 dose)
keto-LCA isomer	C_24_H_38_O_4_	23.6	0.0082	3.1
		25.9	0.032	14.9
		30.3	0.015	3.1
keto-DCA isomer	C_24_H_38_O_5_	10.2	0.027	23.9
		15.3	0.028	23.0
		15.8	0.0018	12.0
		17.8	0.035	13.3
		18.5	0.010	5.3
		20.2	0.018	9.1
		23.0	0.013	2.6
CA isomer	C_24_H_40_O_5_	14.3	0.031	20.0
		18.9	0.014	14.7
GCA isomer	C_26_H_43_NO_6_	6.7	0.043	6.1
		8.4	0.0057	8.9
		9.5	0.0078	14.0
		10.8	0.036	5.6
		11.8	0.021	8.0
GCDCA isomer	C_26_H_43_NO_5_	13.8	0.043	20.5
		19.4	0.0026	8.0
CDCA isomer	C_24_H_40_O_4_	22.5	0.031	60.0
		25.3	0.030	19.5
		26.7	0.010	8.6

* Fold change is derived from comparing peak area ratios of each assigned bile acid with its corresponding IS peak for normalization.

**Table 2:** Tentatively assigned sulfate conjugates with significantly changing peak area ratios in rat plasma between low and high APAP doses.

Bile Acid (Sulf-Isomer)	Formula	*m/z* [M-H]^−^	ppm	RT (min)	*p*-Value	Fold Change* (600/75 dose)
keto-DCA+SO_3_	C_24_H_38_O_8_S	485.2212	−0.5	17.3	0.035	20.7
CA+SO_3_	C_24_H_40_O_8_S	487.2363	−1.7	17.7	0.025	8.2
keto-LCA+SO_3_	C_24_H_38_O_7_S	469.2278	2.7	21.6	0.000085	5.9
CDCA+SO_3_	C_24_H_40_O_7_S	471.2412	−2.1	21.6	0.00025	8.5
		471.2425	0.6	23.9	0.0015	6.3
		471.2405	−3.6	25.3	0.0026	6.9
		471.2411	−2.3	26.5	0.0017	6.5
DHLCA+SO_3_	C_24_H_38_O_6_S	453.2330	3.0	39.1	0.013	8.3
LCA+SO_3_	C_24_H_40_O_6_S	455.2481	1.8	41.8	0.013	14.0

* Fold change is derived from comparing peak area ratios of each assigned bile acid with its corresponding IS peak for normalization. These assignments are tentative and show possible isomeric names only, based on standards present in the bile acid standard mix.

## Data Availability

All data are available in the manuscript, supplementary material, and by request to corresponding author.

## Supplementary Materials

The following supporting information can be downloaded at: https://www.mdpi.com/article/10.3390/ijms24032489/s1.
