# Supplementary material for: Semi-Targeted Profiling of Bile Acids by High-Resolution Mass Spectrometry in a Rat Model of Drug-Induced Liver Injury
Source: Int J Mol Sci. 2023 Jan 27;24(3):2489. doi: 10.3390/ijms24032489 (PMC9917070; doi:10.3390/ijms24032489)
Supplement: Supplementary file 1 [file ijms-24-02489-s001.zip › ijms-2167775-supplementary.pdf]

International Journal of *Molecular Sciences*

MDPI

Supplemental Material

Semi-Targeted Profiling of Bile Acids by High-Resolution Mass Spectrometry in a Rat Model of Drug-Induced Liver Injury

Myriam Mireault, Vivaldy Prinville, Leanne Ohlund and Lekha Sleno *

Department of Chemistry, Université du Québec à Montréal (UQAM), P.O. Box 8888, Downtown Station, Montreal, QC H3P 3C8, Canada
* Correspondence: sleno.lekha@uqam.ca

Table S1. Internal standards chosen for data normalization assigned for standard bile acids

Table S2. Assigned bile acids (present in standard mix) having significant changes in rat plasma treated with low and high APAP dose, with LC-MS data

Table S3. Summary of LC-MS/MS data for unconjugated bile acids from standard mix

Table S4. Summary of LC-MS/MS data for conjugated bile acids from standard mix

Table S5. Summary of LC-MS/MS data for tentatively-assigned bile acid isomers having significant changes between lowest and highest APAP dose

Figure S1. Extracted ion chromatograms of metabolites formed in human S9 fraction with NADPH and PAPS. Diamond represents retention time of the parent molecule. The peaks were scaled and annotated as follows: ❖ increased 5x, * increased 10x and ** increased 20x, for clarity.

Table S1. Internal standards chosen for data normalization assigned for standard bile acids.

Bile Acid	Internal Standard
NDCA, DHLCA, 7-keto-LCA, 12-keto-LCA, APCA, LCA, muro-CA, UDCA, HDCA, CDCA, DCA	DCA-d_4
ACA, CA, 7-keto-DCA, 3-DHCA, UCA, ω-MCA, α-MCA, β-MCA	CA-d_4
GUDCA, GHDCA	GUDCA-d_4
GCDCA	GCDCA-d_4
GDCA	GDCA-d_4
GHCA, GCA	GCA-d_4
TUDCA	TUDCA-d_4
TCDCA	TCDCA-d_4
TDCA	TDCA-d_6
T-ω-MCA, T-α-MCA, T-β-MCA, THCA, TCA	TCA-d_4

Table S2. Assigned bile acids (present in standard mix) having significant changes in rat plasma treated with low and high APAP dose, with LC-MS data.

Bile Acid	Formula	*m/z* [M-H]$^-$	ppm	RT (min)	*p*-value	Fold Change (600/75 dose)
APCA	$C_{24}H_{38}O_4$	389.2701	0.9	28.9	0.0056	5.5
7-keto-DCA	$C_{24}H_{38}O_5$	405.2647	0.1	17.4	0.0046	7.6
muro-CA	$C_{24}H_{40}O_4$	391.2855	0.3	21.7	0.045	34.8
HDCA	$C_{24}H_{40}O_4$	391.2852	-0.5	25.1	0.019	6.4
DCA	$C_{24}H_{40}O_4$	391.2861	1.8	33.6	0.00058	6.0
UCA	$C_{24}H_{40}O_5$	407.2805	-0.5	12.6	0.046	6.3
ω-MCA	$C_{24}H_{40}O_5$	407.2794	-2.2	16.0	0.043	7.1
α-MCA	$C_{24}H_{40}O_5$	407.2797	1.5	17.0	0.030	3.8
ACA	$C_{24}H_{40}O_5$	407.2786	4.2	21.0	0.036	7.9
CA	$C_{24}H_{40}O_5$	407.2796	-1.7	23.2	0.0067	2.4
GHDCA	$C_{26}H_{43}NO_5$	448.3066	-0.6	17.5	0.042	7.5
GDCA	$C_{26}H_{43}NO_5$	448.3065	-0.8	26.3	0.0021	10.6
GCA	$C_{26}H_{43}NO_6$	464.3015	-0.6	16.8	0.0033	3.5

* Fold change is derived from comparing peak area ratios of each assigned bile acid with its corresponding IS peak for normalization.

Table S3. Summary of LC-MS/MS data for unconjugated bile acids from standard mix.

Bile acid	Formula	*m/z* [M-H]$^-$	ppm	RT (min)	Selected MS/MS fragments* (*m/z*)
NCDA	$C_{23}H_{38}O_4$	377.2717	5.2	17.0	-
NUDCA	$C_{23}H_{38}O_4$	377.2699	0.4	28.0	333.2786, **331.2649****, 313.2511
NCA	$C_{23}H_{38}O_5$	393.2637	-2.4	17.0	331.2626, **329.2463**, 275.2036
DHCA	$C_{24}H_{34}O_5$	401.2337	0.9	13.8	383.2193, **331.1925****, 313.1801**, 291.1606, 269.1903, 249.1485**, 215.1446**
DHLCA	$C_{24}H_{38}O_3$	373.2774	6.9	43.6	-
7-keto-LCA	$C_{24}H_{38}O_4$	389.2724	6.8	27.6	-
12-keto-LCA	$C_{24}H_{38}O_4$	389.2723	6.6	28.5	343.2667, **325.2539**
APCA	$C_{24}H_{38}O_4$	389.2693	1.1	29.0	**371.2575**, 243.1745, 169.0876
7-keto-DCA	$C_{24}H_{38}O_5$	405.2654	-1.9	17.2	**361.2738**, 343.2619, 289.2164
3-DHCA	$C_{24}H_{38}O_5$	405.2654	1.9	21.7	**361.2767**, 343.2658, 289.2179,

LCA	$C_{24}H_{40}O_3$	375.2933	7.5	43.4	-
muro-CA	$C_{24}H_{40}O_4$	391.2883	7.5	21.7	-
UDCA	$C_{24}H_{40}O_4$	391.2877	5.9	23.8	-
HDCA	$C_{24}H_{40}O_4$	391.2879	6.4	25.0	-
CDCA	$C_{24}H_{40}O_4$	391.2878	6.2	32.4	-
DCA	$C_{24}H_{40}O_4$	391.2862	-2.1	33.5	355.2641, 347.2948, **345.2783**, 343.2635, 327.2695
IDCA	$C_{24}H_{40}O_4$	391.2858	1.1	39.8	347.2980, **345.2800****, 327.2703
UCA	$C_{24}H_{40}O_5$	407.2828	6.1	12.4	**345.2817**, 343.2673, 327.2708, 325.2568
ω-MCA	$C_{24}H_{40}O_5$	407.2828	6.1	15.8	**405.2672**
α-MCA	$C_{24}H_{40}O_5$	407.2829	6.4	16.8	**405.2675****, 387.2575, 371.2595
β-MCA	$C_{24}H_{40}O_5$	407.2823	4.9	17.9	**371.2578**
ACA	$C_{24}H_{40}O_5$	407.2828	6.1	20.8	**389.2724**
CA	$C_{24}H_{40}O_5$	407.2831	6.9	23.0	363.2938, 361.2769, **345.2817**

* Fragment ions > 1% intensity and ** fragment ions > 5% intensity, most intense fragment in each spectrum bolded. No MS/MS acquired in data-dependent mode for those without any fragment ions listed.

Table S4. Summary of LC-MS/MS data for conjugated bile acids from standard mix.

Bile acid	Formula	*m/z* [M-H]⁻	ppm	RT (min)	Selected MS/MS Fragments
GDHCA	$C_{26}H_{37}NO_6$	458.2568	4.3	8.6	348.1847*, **74.0248****
GHCA	$C_{26}H_{43}NO_6$	464.3037	4.2	14.0	**74.0245****
GUDCA	$C_{26}H_{43}NO_5$	448.308	2.6	16.2	386.3089*, **74.0244****
GCA	$C_{26}H_{43}NO_6$	464.3042	5.3	16.7	402.3051*, **74.0247****
GHDCA	$C_{26}H_{43}NO_5$	448.3081	2.8	17.3	386.3084*, **74.0245****
GCDCA	$C_{26}H_{43}NO_5$	448.3089	4.6	24.9	386.3102*, **74.0243****
GDCA	$C_{26}H_{43}NO_5$	448.3075	-1.5	26.1	404.3166*, **74.0242***
GLCA	$C_{26}H_{43}NO_4$	432.3125	1.3	36.1	388.3234*, **74.0247****
TDHCA	$C_{26}H_{39}NO_7S$	508.2398	4.6	8.0	**124.0076**
T-ω-MCA	$C_{26}H_{45}NO_7S$	514.2868	4.7	9.8	**124.0077**
T-α-MCA	$C_{26}H_{45}NO_7S$	514.286	3.1	10.4	**124.0074**
T-β-MCA	$C_{26}H_{45}NO_7S$	514.2871	5.3	10.9	**124.0074**
THCA	$C_{26}H_{45}NO_7S$	514.2876	6.2	13.7	**124.0086**
TUDCA	$C_{26}H_{45}NO_6S$	498.2917	4.4	16.0	**124.0077**
TCA	$C_{26}H_{45}NO_7S$	514.2879	6.8	16.8	**124.0076**
TCDCA	$C_{26}H_{45}NO_6S$	498.2917	4.4	24.7	**124.0072**
TDCA	$C_{26}H_{45}NO_6S$	498.2916	4.2	26.3	**124.0073**
TLCA	$C_{26}H_{45}NO_5S$	482.2944	-0.4	36.5	**79.9577**

* Fragment ions > 1% intensity and ** fragment ions > 5% intensity, most intense fragment in each spectrum bolded.

Table S5. Summary of LC-MS/MS data for tentatively assigned bile acid isomers having significant changes between lowest and highest APAP doses.

Bile acid	Formula	*m/z* [M-H]⁻	ppm	RT (min)	Selected MS/MS Fragments*
keto-LCA isomer	$C_{24}H_{38}O_4$	389.2694	-0.9	23.6	-
		389.2692	-1.4	25.9	-
		389.2694	-0.9	30.3	**371.2593**, 353.2486, 327.2690, 243.1768
keto-DCA isomer	$C_{24}H_{38}O_5$	405.2644	-0.6	10.2	**387.2553**, 369.2416
		405.264	-1.6	15.3	387.2530**, **369.2424****, 361.2781, 351.2324
		405.2649	0.6	15.8	**387.2526****, 369.2419**
		405.264	-1.6	17.8	-
		405.2638	-2.1	18.5	**387.2532****, 369.2431**
CDCA isomer	$C_{24}H_{40}O_4$	391.2845	2.3	22.5	-
		391.2851	-0.7	25.3	347.2956, **345.2803**, 343.2619, 327.2685
		391.2856	0.6	26.7	347.2973, **345.2799****, 343.2643,
CA isomer	$C_{24}H_{40}O_5$	407.2803	0	14.3	389.2360, 361.2056, 346.2845, **345.2800****, 59.0140
GCDCA isomer	$C_{26}H_{43}NO_5$	448.3059	-2.1	13.8	402.2263, 386.3036, **74.0247****
		448.3063	-1.2	19.4	402.3014, **74.0249****
GCA isomer	$C_{26}H_{43}NO_6$	464.3	-3.8	6.7	**74.0249****
		464.3015	-0.6	8.4	420.3101, **74.0247****
		464.3008	-2.1	9.5	420.3081, 402.3007, **74.0250****
		464.3008	-2.1	10.8	**74.0248****

* Fragment ions > 1% intensity and ** fragment ions > 5% intensity, most intense fragment in each spectrum bolded. No MS/MS acquired in data-dependent mode for those without any fragment ions listed.

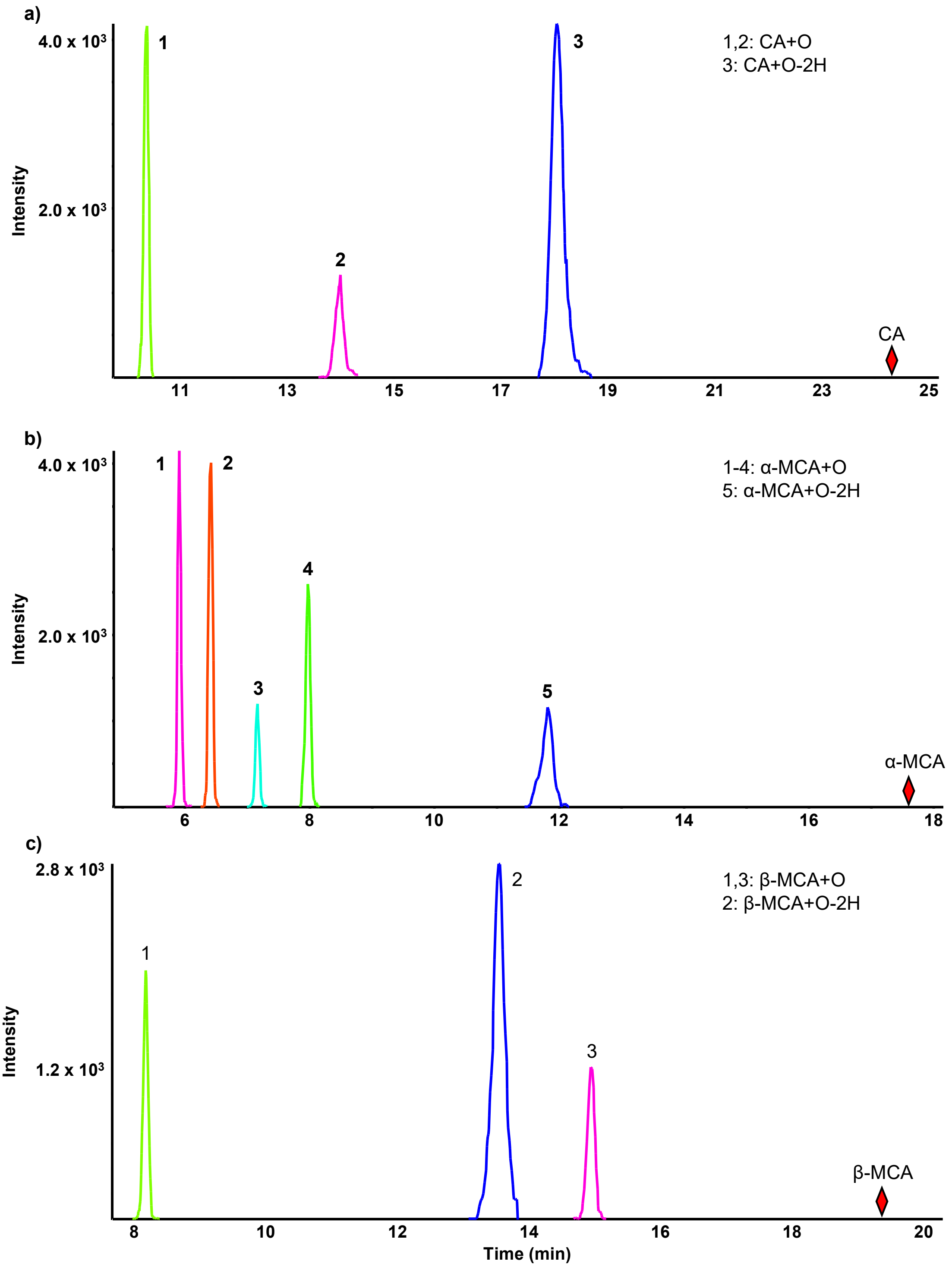

Figure S1 Extracted ion chromatograms of metabolites from a) CA, b) *α*-MCA and c) *β*-MCA formed in human S9 fraction with NADPH and PAPS. Diamond represents retention time of the parent molecule. The peaks were scaled and annotated as follows: ❖ increased 5x, * increased 10x and ** increased 20x, for clarity.

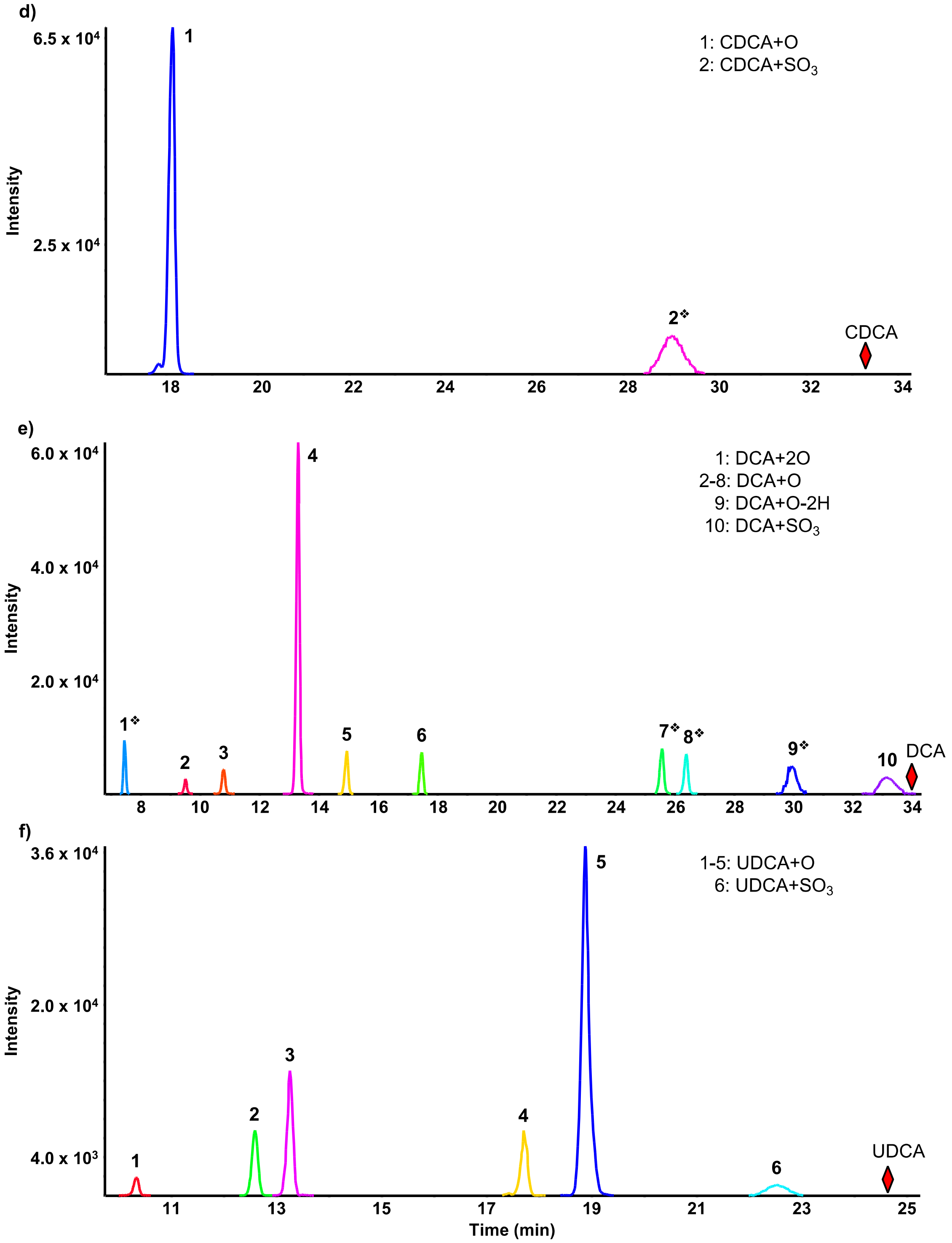

Figure S1 (continued). Extracted ion chromatograms of metabolites from d) CDCA, e) DCA and f) UDCA formed in human S9 fraction with NADPH and PAPS. Diamond represents retention time of the parent molecule. The peaks were scaled and annotated as follows: ❖ increased 5x, * increased 10x and ** increased 20x, for clarity.

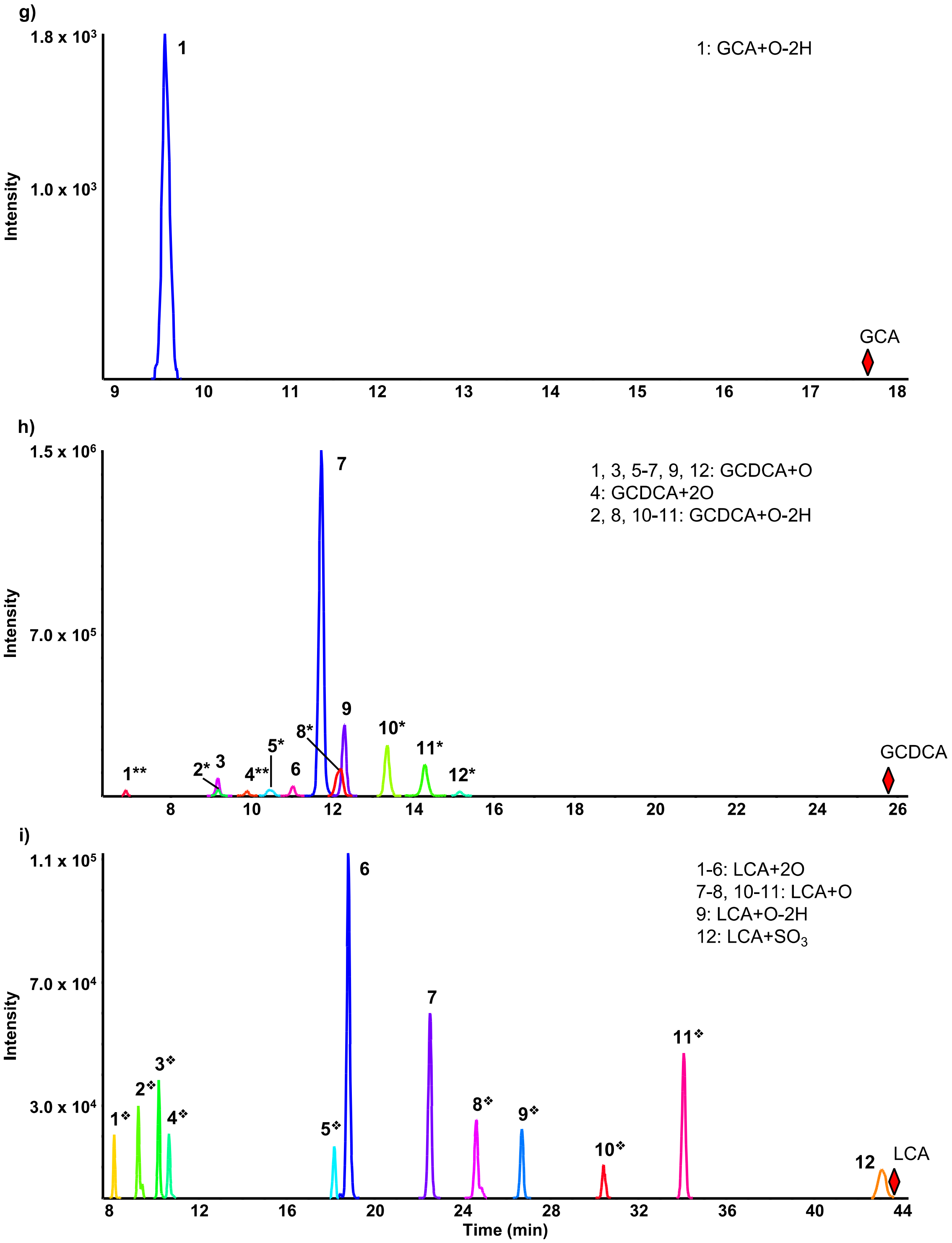

Figure S1 (continued). Extracted ion chromatograms of metabolites from g) GCA and h) GCDCA and i) LCA formed in human S9 fraction with NADPH and PAPS. Diamond represents retention time of the parent molecule. The peaks were scaled and annotated as follows: ❖ increased 5x, * increased 10x and ** increased 20x, for clarity.
